# Injurious Effects of Amalgam Fillings

**Published:** 1894-02

**Authors:** Charles H. Taft

**Affiliations:** Chicago


### International Dental Journal.

Vol. XV. February, 1894. No. 2.

### Original Communications.[1]

### INJURIOUS EFFECTS OF AMALGAM FILLINGS.[2]

BY CHARLES H. TAFT, A.B., D.M.D., CHICAGO.[3]

Mr. President and Brethren of the Harvard Odontological Society,—The invitation to present for your consideration the paper which I had prepared to be read before the Massachusetts Dental Society, upon the invitation of the Secretary to send the Society something for its twenty-eighth annual meeting, and which, as is generally known, was refused a hearing for reasons too puerile to mention here, is especially gratifying to me,—the more so because the invitation comes to me entirely unsolicited, and proves that there is one society at least that is always willing to hear and to discuss anything that claims to be of scientific interest to any member of our profession.

Knowing as I do full well that the possession of convictions is never considered by Harvard men a sin or a crime, and that a man can always be entitled to respect and sincerity of purpose who has the courage to express them, however much others may differ with him, I come to the subject of my paper, and shall explain to the

[1] The editor and publishers are not responsible for the views of authors of papers published in this department, nor for any claim to novelty, or otherwise, that may be made by them. No papers will be received for this department that have appeared in any other journal published in the country.

[2] Read before Harvard Odontological Society, September 28, 1893.

[3] Professor of Dental Surgery and Therapeutics in Hering Medical College Chicago.

best of my ability the reasons why amalgam fillings are often, though not always, a source of evil, considering the subject almost exclusively from the stand-point of those who practise medicine in strict accordance with the teachings of Hahnemann.

That I may not lay myself open to the charge of making dogmatic assertions which rest upon no logical or scientific foundation, permit me to say in the beginning, that no one in the past has been a greater sceptic as to the assertion that amalgam fillings could offer any obstacle to the physician in the treatment of disease than myself. No one, probably, has used amalgam with greater freedom than I have done, or had greater faith and confidence in the efficacy of copper amalgam above all others as the best possible substance to be used in a large percentage of cases, both for the hardening of tooth-substance and the prevention of further decay.

I still hold to my faith in the fact that we have yet to find a substance or material that will better preserve tooth-substance and bone-tissue than pure copper, and I think this fact will be abundantly verified to the satisfaction of any one who will examine the teeth, the bones and crania in many of the skeletons of the ancient tribes of Indians to be found in the Peabody Museum of Archæology and Ethnology at Cambridge. In addition to such verification, we have the results of clinical experience to prove the value of copper as a filling-material.

When, therefore, I make the statement that, earnest as is my desire to save the teeth of my patients, I hesitate to do it with any material which may prove an obstacle to the physician subsequently in the treatment of disease,—however much I may value it as a filling-material,—and that I have discarded the use of amalgam absolutely during the past year of my practice, it may reasonably be assumed that I have taken the trouble to investigate for myself whether the statements of physicians upon the point at issue are correct or not, rather than ridiculing and trying to laugh them away.

What, then, is it that exerts an injurious influence upon the system and proves the obstruction to the proper action of a carefully selected and indicated remedy in the treatment of disease?

That it is the mercury alone which is used to effect an amalgamation of one or more metals in a finely-divided state physicians of both the two great schools of medicine are all agreed: and the claim is therefore often made—more especially by the homœopathic physician—that the indicated remedy given for any diseased condition or disturbance of the vital force often fails in its curative effect when there is apparently no good reason in view for its so doing.

Now, the physician who practises medicine in strict accordance with the teachings of Hahnemann knows that every disease, be it acute or chronic, should yield sooner or later to the action of the one and only one right remedy; and knows still further that no two medicines when mixed together can act in harmony in restoring to the vital force its normal equilibrum any more than two plants growing side by side in the same soil could become associated together without discord,—that is to say, every drug or medicine has a distinct individuality by means of which it alone is capable of successfully attacking a given set of symptoms and restoring the natural harmony among the organs and tissues of any part of the system in which such harmony has been disturbed.

It will be seen, therefore, and can be proven by any one who questions this fact, that the action of an indicated medicine or drug will be only partially successful at times in its curative action, for the reason that there is another drug which is counteracting the effects of the prescribed remedy. In other words, the patient being one who is peculiarly susceptible to mercury is continually getting what are termed *provings* of the drug, and the physician derives about as much satisfaction in the treatment and cure of his patient as does the one whose practice it is to give one or more remedies in alternation, believing that if one does not do the work, the other or both in combination may.

The physician accordingly instructs his patient to go to the dentist and have the fillings removed. The patient, valuing his health, obeys in spite of the assurance generally given that it is all bosh, humbug, or nonsense to rely upon such advice.

What is the result of their removal? Chronic diseases which have hitherto failed to yield to treatment begin at once to yield more quickly and permanently to the action of the medicines. The patient is quick to perceive the improvement in his general health, likewise the family and friends. The action of each new drug which is prescribed as the character of the symptoms change advances the patient steadily on to renewed health instead of having but a temporary effect for the better, only to allow him to slide back to where he originally was, as was the case before the amalgams were removed.

Circumstances and associations the past year have been such as to compel me to investigate for myself, and to frankly acknowledge, as I have not been able to do hitherto, that a marked improvement in the patient's condition almost invariably followed the removal of the amalgams, and that the action of the medicines upon the disturbed vital force was much more marked and effectual following such removal than what it was before.

How, then, it may be asked, do you account for the very frequent desired action of the indicated remedy in the treatment of a patient whose teeth *have* one or more amalgams, while in the treatment of other patients whose teeth are similarly filled the indicated remedy fails? And this brings us to the point I wish to emphasize,—namely, that all persons are not susceptible to the same drugs at all times nor in the same degree,—that is to say, one person may have only to smell of a bottle containing phosphorus, for instance, in the one hundred thousandth or a much higher potency even to get well-marked phosphorus symptoms or provings so called, whereas another person might smell of the bottle all day long without perceiving the slightest ill effect.

Again, one person sleeps for a night in a room the wall-paper of which contains arsenic and gets well-marked symptoms of arsenical poisoning, while another person may sleep in such a room for an indefinite period without perceiving the slightest effect.

Still again, one person passes through or near a bed of poison-ivy with perfect impunity, while another shows soon afterwards unmistakable evidence of ivy-poisoning.

In neither of the above instances is there any of the crude material of the drug taken into the system, and yet no one will deny that there is an altered or perverted condition of the vital force, with symptoms produced by each which could not possibly be mistaken for, or confounded with, those of any other drug.

If it is clear, then, that in neither case is there any of the crude substance to produce the given symptoms, but that the symptoms unmistakably exist, we must face the question, What is it that produces them in either case? and this brings us to the question, What is the vital force, that unseen and untangible something that constitutes the difference between a live man and a dead man? Is it a material substance that can be seen with the eye or felt with the hand? If not, what else can it be but a spirit-like force or substance (as Hahnemann terms it), and which, when becoming disturbed by disease, must be treated with a similar dynamic force rather than with a crude material one?

When we come to reflect and to appreciate the fact that it is this vital force and not the material organs or tissues of the body upon which the action of medicines is to be directed; when we have, too, the fact visibly demonstrated to us that a force which can neither be seen, felt, tasted, or smelt, like that which I have just mentioned, and apparently dormant, can so affect the vital force of a person who is peculiarly susceptible to it as to be manifested in plainly-marked symptoms peculiar to that drug and that alone, then we can discard the idea as worthless that because there is apparently no free mercury in an amalgam filling there can consequently be no dynamic effect to retard the action of any drug which the physician finds it necessary to prescribe.

Whenever we deal with potentized drugs of any kind, so far as their medicinal properties are concerned, we are dealing with forces as subtle and powerful as any of the natural or physical forces which govern the universe.

What matters it how small or in what form the substance exists provided its force, apparently dormant or inert, is unmistakably manifest?

An esteemed member of our profession across the Atlantic has declared that his complete abandonment of high attenuations dates from the time when he discovered that he must either give up this method of treatment or avow his disbelief in the atomic and molecular theories of chemistry. Has any one ever seen a molecule of any material substance? Has any one ever been able to prove that when a molecule has been divided and subdivided for an infinite number of times there has been a limit reached to its further subdivision?

We may search as hard to find with the highest powers of the microscope a single molecule of the material substance of aconite and of many other drugs in the third decimal potency as we may to find one in the forty-five thousandth potency, and yet the *fact* remains that well-marked and distinctive symptoms peculiar to that drug can be derived as well from one as from the other potency when administered to a person in perfect health and who is peculiarly susceptible to aconite.

In the study of embryology and zoölogy the microscope has given us visible demonstration of the fact that all life is cell-life, and that such life is manifested in many of the lowest forms of the vegetable and animal kingdoms by the existence of but a single cell. Conversely, the study of pathological anatomy, which is but a manifestation of perverted life, or what we call disease, has taught us that all disease is cell-disease. The human body being made up of multitudes of cells, and millions upon millions of them so minute as to be well-nigh invisible with the highest powers of the microscope, it is clear that a medicine in order to reach and affect them must be equally minute, for in order to reach them it must enter them, and to accomplish this it must be smaller than they.

A well-known writer has said that "Food, to be appropriated by the body, must go to the stomach and be digested, pass from the stomach and be assimilated; but that medicine, to be effectual, should not travel this route. Digestion would destroy it. It should be so minutely divided that the open-mouthed absorbents swallow it as soon as it comes in contact with the mucous membrane of the mouth. Thus unchanged it enters the circulation.

"A group of cells in a remote corner of the anatomy are hurt and crying out for help. Help is on the road. Over the trunk-lines, past the way-stations, out on the local road, recognized by every road official *en route*, and hurried unerringly to its destination. No need then of further concern or anxiety. If the doctor has selected well, the drug will reach its destination.

"We may not be able to locate the origin of, or always diagnose, disease to our entire satisfaction, but this need not, does not, prevent intelligent and successful treatment of the sick. Every disease or condition of disease will photograph its appropriate remedy, and every remedy true to the picture will accomplish the object designed. In the midst of doubt and uncertainty regarding exact pathological conditions, we can at least be sure that disease is not an entity; that it cannot be expelled by emetics, cathartics, diuretics, or diaphoretics; that it is wrong life, perverted life, inharmonious, discordant life; and that, while it may be coaxed back into tune and harmony, it will not, cannot, be coerced."

The subject at issue has to deal so closely with the dynamic influence of drugs that I find it difficult to attempt to express myself clearly to those who do not fully understand the meaning of the term and manner in which this spirit-like force (the real medicinal property) of the drug, as Hahnemann terms it, is evolved or set free when the drug is potentized or dynamized.

To all such persons I would suggest that a careful perusal of one of Hahnemann's works, entitled "The Organon, or Art of Healing," will throw much light on many things in my paper which may otherwise seem very obscure.

It is a well-known fact that every drug is capable of producing well-marked symptoms which are characteristic of it when administered either in the highest or lowest potencies to persons in health.

Let all who are inclined to doubt this statement take the trouble to verify it by actual experiment instead of prematurely ridiculing it, as many no doubt will be inclined to do, for I do not expect any one to accept the truth of any statement I may make upon my own *ipse dixit*, or with that "happy blind faith of childhood" which some of my friends across the water declare they no longer possess. When we have proved this to our satisfaction we can then accept the fact that because the mercury has entered into the formation of a chemical compound it has none the less preserved the dynamic force which it and all other drugs possess.

The doctrine of the conservation of energy, which declares that force is never destroyed whatever its changes in manifestation may be, is as applicable to drugs and their dynamic action, even though the force is shut up in an amalgam filling and *apparently* inert, as it is to a simple piece of charcoal, which needs but a spark to make more manifest the hidden energy stored up in its component parts, and upon the liberation of which may show itself subsequently in manifold forms, such as heat, vapor, gases, and all the compounds both of organic and of inorganic life.

A man who does not thoroughly understand and accept this doctrine as one of the established laws of the universe will not be likely to understand the action or, in other words, the energy of drugs when potentized and the laws which govern it; for the energy which lies hidden in the mercury of an amalgam filling is none the less potent and subject to these same laws than it is either in its crude state or as a component part of some compound other than an amalgam filling.

I have heard men in our profession deny with the greatest positiveness, without attempting to offer any reason for their assertion, that there can ever be any possible injurious effect or influence produced upon the system by amalgam fillings.

Such men I believe in all sincerity are entirely unaware that mercury is one of the most poisonous of drugs and one of the most difficult to eliminate from a system which has once been susceptible to its toxic effects. There is surely no other drug, poison, or disease, not even syphilis itself in its worst form, that becomes a more difficult matter for the physician to eradicate from the system. Being one of the most deep-seated and deep-acting drugs, its physiological effects often come under our observation within the field of our own special work.

The least we can do in getting at the truth of the point at issue between the two professions is, first, to study the action of the drug both as to its physiological and medicinal properties; second, to study carefully the dual action of drugs in general; third, to study the laws which govern such action; fourth, the proving of potentized drugs upon one's self with a view to becoming satisfied whether or not there *is* that dynamic action in them and possessing actual curative properties which all who have faithfully studied them know they actually possess; and, fifth, the bearing of these laws upon the question we are considering.

When I assure you that such has been my endeavor the past year that I might be able to speak without prejudice in writing a paper upon this subject, you will readily understand that I come before you not for the purpose of stirring up personal controversies and seeking besides the disruption of societies, as it was argued I would do had my paper been granted a hearing before the Massachusetts Dental Society, but rather from an earnest desire to contribute my share of whatever may be of help or interest to any man in our profession.

It may be asked, Why are not the cement fillings equally injurious, considering their composition?

I reply that there is, in both cement and gold fillings, the same kind of dynamic force, but that it is not of such a poisonous character, nor does it act in either case as such a powerful obstruction to the action of indicated remedies as is unquestionably true of mercury.

I may say right here that I have one patient who is so susceptible to aurum that whenever a new gold filling is inserted in any of her teeth an ulcer invariably appears within twenty-four hours upon the mucous membrane immediately about the tooth and upon the tip of the tongue from the constant touching of the tongue against the filling,—the taste of the metal being as plainly evident to the patient as is that of the copper in a copper amalgam filling to patients peculiarly susceptible to cuprum. A glance at the homœopathic materia medica will reveal the ulcerations I have alluded to under the symptoms obtained from provings of aurum.

Among my patients are many sent to me suffering from chronic diseases and complaints of various kinds, with instructions from the physician to remove all amalgam fillings. The scepticism of a large majority of such patients, supplemented by that of their families and friends, that a removal of the fillings would result in any visible improvement in the patient's health has been generally equal to what my own want of faith until recently was, that there was either truth or sense in the statement made by physicians, and I am frank to admit that no person who was familiar with the patient's condition before the fillings were removed, and by a subsequent following of the case to notice the effect, if any, of such removal, could be more amazed than I have been at the improvement which has invariably followed.

Did time permit I should be glad to speak in detail of many cases where the action of the physician's medicines subsequent to the removal of amalgams was all that he knew it was capable of doing and should do in sending his patient on to a speedy improvement in health.

These cases are all a matter of record, and if any one desires proof that I am making statements that cannot be fully substantiated, I shall be happy to give him the names of such patients, to whom he can personally write for all the information he desires.

Therefore, if my statements appear to any one at all dogmatic, let it be remembered that I have made them fearlessly, and with the hope that all who take exception to them will remember that they have not only the same opportunity to verify them that I have had but that it is their *duty* to do so.

The opposition to amalgam fillings, gentlemen, is one that has come to stay. Let no one deceive himself on this point or try to laugh it away, for it is by no manner of means confined to "a few physicians practising in Boston," as a former president of the Massachusetts Dental Society declared so positively at the twenty-seventh annual meeting of that Society. A little investigation would have shown him that it covers a territory extending from Maine to California, and from Texas to the extreme north of Canada, while a visit to the shores across the Atlantic would have shown to him the same unyielding opposition.

It will not be understood from what I have said that I believe amalgam fillings in every case offer any obstacle to the physician in the treatment of disease, and consequently have any evil effect upon the system, for it is only when a person whose teeth are filled with amalgam is peculiarly susceptible to mercury that medicines will not have their proper curative effect; but the fact that a person may be susceptible to-morrow if not to-day should make us recognize the duty of abandoning absolutely the use of amalgam so far as it is possible to do so.

Speaking upon the subject we are considering, the statement was made by a member of the Massachusetts Dental Society at the twenty-seventh annual meeting that he did not wish to be compelled to ask, when treating a patient's teeth, "Who is your medical adviser?" and to act on the doctor's judgment instead of his own.

Would it not be well when a patient is sent to us with instructions from the physician to remove all amalgam fillings from the teeth of his patient to courteously comply with the request, at the same time taking pains to follow the case after it has left our hands and note whether there is any improvement or not? If there is, it will at least set an honest man to thinking, and prompt him to follow a line of study similar to the one I have suggested. If there is no improvement, then he can at least have the satisfaction of going to the physician with a request for the reasons why his medicines then fail to do what he claimed they would in restoring health to his patients.

Health is one of the dearest things of life, and no one sufficiently prizes its value until he has been deprived of it. Among my patients are those who have not known for years what a return of it meant to them until it began to be gradually restored to them after I had conscientiously and painstakingly removed every particle of amalgam from their teeth, with as earnest a desire to prove that physicians were either wholly right or wholly wrong as to do what lay in my power in helping to bring to the patient what he was most in search of.

Therefore I feel that so long as we as dentists are content to stand aside and obstinately ridicule and contradict statements made by physicians eminent not only for their skill in successfully combating disease, but for their scholarly and scientific attainments as well, before we have patiently investigated the matter for ourselves and obtained some positive knowledge, so long shall we be in a humor to discuss the question before us without profit to ourselves and without the desire even of acquiring any new scientific truth.

One fact or principle evolved by both inductive and deductive processes of reasoning, and plainly demonstrated by actual experiment so there is no way of getting around its acceptance, is worth a thousand idle or thoughtless assertions from you or me which prove nothing, having nothing but an individual opinion back of them on which to rest.

Due regard for your patience in listening to my paper will permit me to allude to but one of the most interesting cases I have ever followed, where some twenty or more amalgams were removed from the teeth of a patient who had been almost an invalid, suffering from a complication of troubles for many years, and whose testimony is but that which has been expressed to me by other patients, often in much stronger terms and supplemented by the severest condemnation of amalgam fillings.

Upon my request recently that he give me a brief statement of his case he writes as follows: "Until four years ago I had been all my life under allopathic treatment, taking large quantities of medicine and being, I have no doubt, much of the time drug sick. For nearly four years I have been under homœopathic treatment, and while for the first two years I experienced great relief, much greater than ever before in my life, still the improvement was not so great or so lasting as my physician wished, and at his request I came, as you know, to you to have all my amalgams removed. Although the suffering was great I can truly say I am glad it was done, as my health has improved much faster since then, and I have every reason to believe it is because I am free from the injurious dynamic influence of the mercury contained in the amalgams. I have now better health than I have had for years, and it is rapidly improving."

Relative to the circumstances which have culminated in the reading of my paper before a society other than the one for which it was originally prepared, I may be permitted to say a few words in closing not entirely irrelevant to the subject before us.

In his response to the toast "The World's Columbian Dental Congress," at a recent meeting of the Central Dental Association of Northern New Jersey, the president of that Congress is reported, in the July issue of the International Dental Journal, to have given utterance to the following words: "*Every man who has a thought of value, a fact which is of interest to his fellow-practitioners, will have an opportunity to present that thought or that fact to that Congress.*"

In view of the determined opposition which the distinguished gentleman himself has in the past maintained towards any one desiring to come before the Massachusetts Dental Society with a paper bearing the title of the one under consideration, and the opportunity given by that Congress to every man to act in accordance with the most liberal interpretation of that sentiment, may it not be within reason to hope that, forgetting the childish, not to say discourteous, action which the Massachusetts Society took a year ago towards one of Boston's most scholarly physicians, and, more recently, the same action towards one of its own corresponding members in good standing, the day will yet come when an opportunity will *always* be cheerfully given to you or to any man who has a thought of value, a fact which is of interest to his fellow-practitioners, to present that thought or that fact to that society and under whatever *title* he may see fit to select?

Assuming that not the least of the objects and lessons of the recent Congress was the giving to every man the fullest encouragement to that kind of scientific investigation which leads to the discovery of truth, the granting to every man the privilege to bring before any society the results of such investigations, and at the same time the cultivation of professional courtesy among members of the same calling, you will, I am sure, join with me in the hope that that day is rapidly disappearing when such a spirit of mediæval intolerance as that so recently exhibited by your State Society will ever again be tolerated by any society that claims to be scientific.

And now let me say in conclusion, that if my paper stirs any thoughtful mind to make a careful and conscientious investigation of this matter before indulging in sweeping assertions and slurs upon our friends of the medical profession for the sturdy maintenance of convictions which are born of experience and founded upon well-established laws, the time you have given to its hearing will have been neither unwisely nor unprofitably spent.

